# Integrative taxonomy of *Cedrela* (Meliaceae) leads to the recognition of a new species (*C. tamaulipana*) and the reinstatement of *C. saxatilis*

**DOI:** 10.1371/journal.pone.0329846

**Published:** 2025-09-17

**Authors:** Sergio Ignacio Gallardo-Yobal, José Antonio Vázquez-García, Alma Rosa Villalobos-Arámbula, Teresa Terrazas, Hilda Palacios-Juárez, Dolores Marina Barragán-Reynaga, Martin Berrones, Alondra Salomé Ortega-Peña, Gerardo Hernández-Vera, Viacheslav Shalisko

**Affiliations:** 1 Instituto Tecnológico Superior de Zongolica, Tecnológico Nacional de México, Zongolica, Veracruz, México; 2 Laboratorio de Ecosistemática, Instituto de Botánica, Departamento de Botánica y Zoología, Centro Universitario de Ciencias Biológicas y Agropecuarias, Universidad de Guadalajara, Zapopan, Jalisco, México; 3 Laboratorio de Genética, Ecosistemática Molecular y Funcional, Departamento de Biología Celular y Molecular, Centro Universitario de Ciencias Biológicas y Agropecuarias, Universidad de Guadalajara, Zapopan, Jalisco, México; 4 Departamento de Botánica, Instituto de Biología, Universidad Nacional Autónoma de México, Coyoacán, Ciudad de México, México; 5 Departamento de Madera, Celulosa y Papel, Centro Universitario de Ciencias Exactas e Ingenierías, Universidad de Guadalajara, Zapopan, Jalisco, México; 6 Unidad Académica Multidisciplinaria Mante, Universidad Autónoma de Tamaulipas, Cd. Mante, Tamaulipas, México; 7 Departamento de Producción Forestal, Centro Universitario de Ciencias Biológicas y Agropecuarias, Universidad de Guadalajara, Zapopan, Jalisco, México; Kerman University of Medical Sciences, IRAN, ISLAMIC REPUBLIC OF

## Abstract

A new species of *Cedrela* (Meliaceae) is described and illustrated from recently discovered populations at Rancho del Cielo Biosphere Reserve, Tamaulipas, Mexico. The newly proposed species is morphologically close to *C. monroana* but differs from the latter in having a shorter habit, thinner terminal twigs, shorter space between leaf pairs along the rachis, shorter petiolules, smaller leaflets, smaller leaflet length-to-width ratio, less numerous secondary leaflet veins, shorter panicles, and yellowish green flowers, broadly obovoid to pyriform fruits, with valves opening at least at an angle of 20 degrees and brown mature capsules with prominent lenticels on valves. We provide a key to the Mexican species of *Cedrela* including the closely related *C. monroana*. Latitudinal differences also support the setting aside of the proposed species, which is the most septentrional among its close relatives within *Cedrela*. Bayesian inference and maximum likelihood analyses of nuclear (ITS) and chloroplast (*accD*, *matK*, *rbcL*, *trnH-psbA*, *psbB-T-N*, *rpl16*, *rpoB*, *rpoC1*, *trnS-G*) DNA sequences of 19 taxa of *Cedrela* plus 3 from related taxa, place the proposed new species within a clade including Mexican & Central American species. Based on morphological and phylogenetic evidence, we propose the reinstatement of *C. saxatilis* as a valid species, previously treated as a synonym of *C. oaxacensis*.

## Introduction

*Cedrela* P. Browne is a New World genus of the tribe Cedreleae DC. (Meliaceae, Sapindales) [1] consisting of 21 species distributed from northeastern Mexico to northern Argentina and Paraguay [2–10]. The sister genus of *Cedrela*, *Toona* (Endl.) M. Roem.*,* exhibits a Paleotropical distribution, with only five species in India, Indo-China, Malesia, and Australasia [11,12]. Cedreleae and its two genera are well characterized morphologically, and molecular phylogenetic analyses support the monophyly of the tribe [13–15], whose geographic origin, based on fossil evidence, is inferred to be in the Northern Hemisphere [5,6,16].

*Cedrela* appears in the fossil record of temperate North America from the Early Eocene up to the Middle Miocene, but it became extinct there in the late Miocene [6]. Phylogenetic and biogeographical studies of *Cedrela* are important for understanding current patterns and processes of diversity within the genus; however, much of this diversity still awaits to be discovered and described. A correct taxonomic identification of sampled individuals is essential for a scientifically sound interpretation of phylogenetic and biogeographic reconstructions, but also to appropriately inform management and conservation strategies, especially for economically important trees like *Cedrela* [7,17].

The taxonomy of *Cedrela* is far from complete and the number of new taxa is expected to increase. Four new cryptic species within the *C*. *odorata* complex are described in the monograph of *Cedrela* by Pennington & Muellner [7]. Likewise, four other species were recently described [8–10,18]. Cavers et al. [3] and Finch et al. [19] also report cryptic species within the *C. odorata* complex in Central and South America.

Widely distributed species often result in polyphyletic groups, entailing several cryptic species, partly due to the influence of environmental and geographical factors as drivers on diversification [3]. For instance, molecular data [5] do not support the traditional concept [20] for *C. odorata* L. as a widely distributed species from northern Mexico to southern Brazil and the Caribbean [7,21]. Some populations of *C*. *odorata* display morphological differentiation in less conspicuous characters such as seeds and seedlings, which are used to subdivide the species [22]. An integrative approach that includes all possible sources of informative characters is necessary to reveal the additional cryptic diversity within the genus. Further fieldwork and additional molecular sampling are also needed to test the species identity of numerous populations within the *C. odorata* species complex beyond the Caribbean region.

In Mexico, there are seven known species of *Cedrela*: four endemic species (*C*. *discolor* S.F. Blake from Durango in northwestern Mexico, *C. dugesii* S. Wats. from western Mexico, *C. saxatilis* Rose and *C*. *oaxacensis* C. DC. & Rose from southern Mexico), and three species with a broad distribution (*C. salvadorensis* Standl. and *C. tonduzii* C. DC., both mostly confined to Mesoamerica, and *C*. *odorata* s. str. from Mexico to the Caribbean region) (Table 1).

**Table 1:** Differences between *Cedrela tamaulipana* and its phylogenetically closely related species, including sympatric *C. odorata.* Key differences between *C. tamaulipana* and *C. monroana* are highlighted in bold font.

	*C. discolor*	*C. dugesii*	C. monroana*	*C. oaxacensis*	*C. odorata*	*C. salvadorensis*	C. saxatilis**	*C. tamaulipana*	*C. tonduzii*
Types and additional material examined (Full information in S2 File)	*Palmer 184* (HT: US; IT: CM, F, GH, K, NY, MO, P, S).	*Dugès s.n.* (HT: GH; IT: A, GH, NY, P); *Machuca 1275* (USF).	*Monro & Alexander 3081* (HT: BM, IT: B, BM, K, LAGU, MO, NY, US); *Martínez 265* (B, BM, LAGU, MEXU, MO); *Monro, Monterossa & Carballo 3789* (B, BM, ITIC, LAGU, MO); *Galan 6026* (B, LAGU, MO).	*Andrieux 483* (HT: K, IT: G-DC); *Pringle 4802* (A, B, BM, CM, F, GH, MEXU, MO, PH, S, US).	Plate 10 (Browne 1756); *Franck et al. 3879* (USF). *Berrones-Morales 22* (IBUG) & *23* (IBUG),	*Calderón 1007* (HT: US; IT: US); *Martínez 28* (MEXU); *Conzatti 3922* (US).	*Rose & Painter 6950* (HT: US; IT: GH, K, MEXU, NY, US, VT); *Beitel s.n.* (OSC).	*Berrones-Morales 1* (HT: UAT; IT: IBUG); *Berrones-Morales 26* (IBUG) & *27* (IBUG), *Gallardo-Yobal 145* (UAT).	*Tonduz 11945* (HT: CR; IT: BM, BR, G, GH, K, NY, US).
Tree height (m) Diameter (cm, dbh) Trunk Bark	? ? ? ?	10.0–12.0(–15.0) 60.0 Somewhat tortuous. Smooth to fissured, grayish.	**Up to 23.0** 60.0 ? Fissured, **obscure grayish brown**.	8.0–10.0 20.0–30.0 Somewhat tortuous. Smooth, reddish.	20.0–30.0(–40.0) 170.0 Straight. Longitudinally fissured, grayish to brown-reddish.	Up to 15.0 30.0 Contorted. Fissured, scaling, and grayish.	4.0–7.0 ? ? Smooth reddish bark	**9.0–10.0** 20.0–100.0 Extremely tortuous Longitudinally deeply fissured, corky, **blackish**.	Up to 50 150.0 ? Fissured and scaling in small regular plates, obscure and grayish-brown.
Leaf length (cm), including petiole	33.5–42.0	10.0–55.0	45.0–55.0	15.0–35.0	15.0–55.0	15.0–25.0	20.0-40.0	40.0–60.0	40.0–70.0
Number of pairs of leaflets	4–8	3–7	7–11	3–7	4–15	4–8	5–7	6–9	5–12
Leaf indumentum Adaxial/ Abaxial	Glabrous/ Pale greyish-white densely appressed puberulous	Glabrous/Domatia on axils of secondary veins	Glabrous	Glabrous or midrib puberulous/ Pale tomentose or villose: hairs crisped, spreading, and whitish	Usually glabrous or rarely puberulous/ Domatia nearly always present	Sparsely pubescent/ Softly pubescent with crisped hairs	Glabrous or slightly puberolous	Glabrous	Glabrous or midrib puberulous/ Sparse to dense pale hairs
Petiolule length (mm)	4.0	7.0–13.0	**3.0–9.0**	2.0–4.0	8.0–10.0	2.0–3.0	2.5–5.0	**1.0–2.0**	1.0–3.0
Leaflet size (cm) Length/width ratio	14.0 × 3.3–15.0 × 3.3 4.4–4.5	10.0 × 3.6– 14.5 × 5.3 2.7–2.8	**14.0 × 5.0–19.0 × 6.9** **2.7–2.8**	5.0 × 3.0–9.4 × 4.5 1.7–2.1	8.0 × 2.5–15.0 × 4.5 3.2–3.3	6.5 × 3.6–13.0 × 7.0 1.8–1.9	10.0 × 4.5–15.4 × 6.7 2.2–2.3	**7.0 × 3.6–11.0 × 4.7** **1.7–2.4**	14.0 × 5.0–20.5 × 8.0 2.8–2.5
Leaflet shape Leaflet apex	Lanceolate to oblong-lanceolate. Narrowly attenuate.	Ovate to ovate-triangular. Long acuminate.	Broadly lanceolate to elliptic-lanceolate. Narrowly attenuate, to acuminate.	Ovate to oblong-lanceolate. Acute to short acuminate.	Lanceolate to oblong-lanceolate. Acute to short acuminate.	Broadly oblong to elliptic to ovate. Obtuse to rounded, obtusely cuspidate.	Broadly lanceolate. Acute to strongly acuminate.	Elliptic to lanceolate. Acute to acuminate.	Oblong-lanceolate. Narrowly acuminate
Secondary veins	14	10–13	**15–19**	10–16	9–14	5–11	15–16	**9–11**	15–20
Panicles Length (cm) Flowered	24.0–28.0 Dense	(5.0–)10.0–18.0 Dense.	**40.0–60.0** Lax.	20.0–35.0 Lax.	15.0–30.0 Lax.	10.0–25.0 Dense.	30 or more	**27.0–30.0(–35.0)** Lax.	25.0–30.0 Dense.
Angle (in degrees) of panicle branches.	≈90	45	≈45–90	≈90	≈90	≈90	30-40, reflexed	45	30–45
Degree of the union of the sepals (mm)	0.7	1.0–1.5	1.0–1.5	1.5–2.0	1.5–2.5	1.0–1.5	?	0.5–1.0	2.0–2.5
Margin adnation of the petals/portion fused to the androgynophore	Lower. ⅓.	Most of their length at anthesis. ?	+/-Adnate at anthesis. ½.	Most of their length. ½ or ⅓.	Most of their length. ⅓ or ½.	Most of their length. ⅓.	? ?	Most of their length at anthesis. ½ or ⅓.	Most of their length. ⅓.
Flower color	Yellowish green	Greenish with red	**Pale pinkish to deep reddish-purple**	Pinkish	White greenish	Pinkish	Purplish	**Green yellowish**	White greenish
Fruit length (cm) Capsules shape	? ?	(2.5–)4.2 Ellipsoid to obovoid	3.5–4.0 **Ellipsoid to slightly obovoid**.	3.5–4.0 Erect, oblongoid cylindroid to obovoid.	3.0–4.5 Broadly ellipsoid to obovoid.	8.0–14.0 Obovoid to oblong.	2.0 Narrowly ellipsoid?	3.0–3.5 **Broadly obovoid to pyriform**.	5.0–8.0 Ellipsoid to slightly obovoid.
Carpel valves: Thickness (mm) Position Lenticels	? ? ?	1.0–1.5 Curved inward, with few or inconspicuous lenticels.	**1.0–1.5** **Closed (+/-), not reflexing, inconspicuous lenticels if any.**	1.0 Nearly straight, with numerous lenticels.	1.5–2.0 ? With evident white lenticels.	3.0–5.0 Diverging, with large lenticels.	? ? Absent	**4.0–5.0** **Curved inward, with prominent ocher lenticels**.	2.0–4.0 Diverging, with pale lenticels.
Flowering	May	Mar.–Jun.	**Aug.–Oct.**	Jul.–Sep.	May–Aug.	Dec.–Feb.	Sept.	**May–Jun**.	Mar.–Aug.
Latitude range	25°37’N	15°22’N–18°11’N	**13°30’N–14°35’N**	19°26’N–20°41’N	7°37’N–27°30’N	9°00’N–22°14’N	Ca. 16°00’N–19°00’N	**23°00’N–23°10’N**	8°00’N–18°00’N
Elevation range (m a.s.l.)	1400–2000	1500–2400	**450–1600**	580–2200	400–1200	350–1400	1500–2600	**1000–1100**	1000–2500
Distribution	Mexico: Durango.	Mexico: Jalisco, Michoacán, Guanajuato and Querétaro.	**El Salvador & Guatemala:** Caldera volcano.	Mexico: Guerrero, Morelos, Oaxaca, Puebla and Chiapas.	Mexico, Central America and The Caribbean	From Jalisco, Mexico to San José, Costa Rica along the pacific slopes.	Mexico: Oaxaca and Morelos.	**Mexico**: Tamaulipas.	From Oaxaca and Chiapas, Mexico to Panama.
Biogeographic provinces (following Morrone [45])	Sierra Madre Occidental.	Southern Sierra Madre Occidental, Transmexican Volcanic Belt.	**Chiapas Highlands**.	Balsas Basin, Sierra Madre del Sur, Pacific Lowlands, Chiapas Highlands.	All provinces of Mexican transition zone except Sierra Madre Occidental and Transmexican Volcanic Belt; provinces of Antillean Subregión,Brazilian Subregion, Chacoan Subregion.	Trans Mexican Volcanic Belt, Pacific Lowlands, Sierra Madre del Sur, Chiapas Highlands, Veracruzan, Puntarenas-Chiriquí.	Trans-Mexican Volcanic Belt.	**Sierra Madre Oriental**.	Pacific Lowlands, Sierra Madre del Sur, Chiapas Highlands, Veracruzan, Mosquito, Guatuso-Talamanca, Puntarenas-Chiriquí.
Vegetation/ soils	Between tropical dry forest & pine-oak forest/?.	Tropical dry forest/ in volcanic soils.	Tropical subdeciduous forests, often in coffee plantations/?	Pine-oak forest/ in volcanic soils.	Tropical subdeciduous forests.	Tropical dry forest / Limestone outcrops.	?/ Volcanic rocks	Tropical subdeciduous forests/ in calcareous soils.	Wet montane forests/?

*Cedrela monroensis was corrected to *C. monroana,* due to its improper epithet termination, under the ICN (Shenzhen Code) Art. 32.2 and 60.8(c).

**Combined morphological and molecular evidence, in this work, supports the status of *Cedrela saxatilis* as a distinct species, separate from *C. oaxacensis*.

Using an integrative approach based on morphological, anatomical, and ecological data, here we describe a new species of *Cedrela* from eastern Mexico, endemic to the northernmost distributional range of the genus. Additionally, we use nuclear and cpDNA sequences to assess its phylogenetic position. Based on morphological and phylogenetic evidence, we propose the reinstatement of *C. saxatilis* as a valid species, previously treated as a synonym of *C. oaxacensis*.

## Materials & methods

### Field work and populations studied

Access to the field sites and sampling was granted by the Municipal Authority of Gómez Farías, Tamaulipas, Mexico (permits PM/SA/02/2022 and PM/SA/164/2022). The populations studied occur in the Sierra de Gómez Farías, in Tamaulipas, Mexico.

Field work was conducted between February and August from 2018 to 2023, coinciding with the flowering period of the species. A total of 48 individuals of *Cedrela* sp. were recorded within the El Cielo Biosphere Reserve. The highest concentration of specimens was found in the type locality population, where 23 adult trees were identified.

### Morphology

The morphological description and the illustrations were based on both fresh and herbarium material (Table 1). Numerous extra specimens of Mesoamerican *Cedrela* spp. were examined in major pertinent herbaria (B, BM, EAP, F, IBUG, K, LAGU, MEXU, MO, OSU, NY, UAT, US, USF), seeking a broad understanding of all closely related taxa as well as dozens of specimens of the geographically close and widely distributed *Cedrela odorata* (including *Berrones-Morales 22 & 23*, at IBUG, for this study). Electronic images of the type material of all eight taxa from Table 1 were consulted at the websites of some of the aforementioned herbaria. The herbarium acronyms follow Thiers [23]. Measurement of morphological characters for the new species was performed on 12 adult trees, of which 6 had floral structures.

Leaf description and terminology of reproductive structures follow Radford et al. [24] and Pennington & Muellner [7]. The diagnosis and comparative Table 1 primarily involved diagnostic characters following Pennington & Muellner [7]. We added some novel characters because they showed variability and proved to be of diagnostic value (non-overlapping), at least between two species or even among several closely related taxa; namely: (1) leaflet length to width ratio, (2) petiolule length, (3) panicle length, (4) visual flower density, (5) angle of primary branches in panicles, (6) flower color and (7) thickness of capsule valves.

Using several non-overlapping diagnostic characters (Table 1), we document substantial morphological differences between *Cedrela* sp. from Tamaulipas and eight *Cedrela* species from Mexico and Central America, including the widely distributed *C. odorata* also occurring close to the type locality of the proposed new *Cedrela* species described here.

### Wood anatomical comparison

Two wood samples of *Cedrela* from cut-down adult trees were obtained for anatomical analysis; one from the “red cedar” initially treated as *C*. aff. *odorata* (*Gallardo-Yobal 9*, UAT)— which after the interpretation of results turned out to be *C. odorata* s. str.—and another one from the locally named “black cedar”, also called “walnut cedar”, *Cedrela* sp. from Tamaulipas (*Gallardo-Yobal 10*, UAT); both samples from Rancho El Cielo Biosphere Reserve. Transverse, tangential and radial sections (20 µm thick) were cut with a sliding microtome. The sections were gradually dehydrated with an ethanol concentration gradient (50%, 70%, and 96%), stained with safranin and mounted in temporal slides in glyceryl-water to perform observations. The presented data follow the recommendations of the IAWA Committee [25] and are compared with those from the species of *Cedrela* available in the database InsideWood [26] as well as a specimen stored as *C. odorata* from the wood collection of the Institute of Biology, UNAM (catalog number XP-224).

### DNA extraction, PCR amplification, and sequencing

Total genomic DNA was extracted from silica-gel dried material (leaf fragments) from the five specimens of *Cedrela* from Tamaulipas (S1 File), using a modified salt-extraction method with 1% PVP [27]. The nuclear genomic region ITS (internal transcribed spacer), and seven chloroplast genomic regions (*accD*, *matK*, *rbcL*, *psbB-T-N*, *rpl16*, *trnH-psbA*, and *trnS-G*) were amplified by PCR reactions performed with 1 × KCl reaction buffer, 5 mM MgCl_2_, 0.2 mM of each dNTP, 0.5 μM of each primer, 0.5 U of Taq polymerase, 0.2 μg/μL of Bovine Serum Albumine (BSA), and 1 μL of DNA template (10 ng/μL) in a 50 μL final volume. For the ITS we used primers F1-ITS and R1-ITS [28]; for the *rbcL* genomic region, we used primers rbcLa-F [29] and rbcLa-R [30]; for *trnH-psbA*, primers psbA3_f [31] and trnHf_05 [32]; for *matK*, primers XF [33] and MALVR1 [34]; for *psbB-T-N*, primers psbH and psb_B [35]; for *accD*, primers accD-IGSF and psaI-IGSR [36]; for *trnS-G*, primers trnSGF and trnSGR [37]*;* for *rpl16*, primers rpl6_1F and rpl16_3R [37]. PCR cycles were carried out using the following thermal profile for *rbcL* and *trnH-psbA*: 95 °C for 5 min, 35 cycles at 95 °C for 48 s, 55 °C for 30 s, 72 °C for 30 s, and a final extension at 72 °C for 10 min. For the *matK* region: 94 °C for 4 min, then 35 cycles at 94 °C for 1 min, 48 °C for 1 min, 72 °C for 1 min, and a final extension at 72 °C for 10 min. For the *psbB-T-N*, *accD*, *trnS-G, and rpl16* regions: 94 °C for 4 min, then 34 cycles at 94 °C for 1 min, 52 °C for 1.5 min, 72 °C for 2 min, and a final extension at 72 °C for 10 min. PCR products were cleaned using Illustra GFX columns (GE Healthcare); purified products were sent to the University of Arizona Genetics Core (UAGC) for DNA sequencing. Contigs from forward and reverse reads were assembled and annotated in Geneious 8.1.9 (Biomatters Ltd., Auckland, New Zealand) and deposited in GenBank (see S1 File for accession numbers).

### GenBank survey and sequence alignment

To assess the phylogenetic placement of *Cedrela* sp. from Tamaulipas, we retrieved DNA sequences from GenBank representative of the tribe Cedreleae, also including sequences from more distantly related genera; cpDNA sequences from three coding (*accD*, *matK*, *rbcL*) and six non-coding (*trnH-psbA*, *psbB-T-N*, *rpl16*, *rpoB*, *rpoC1*, *trnS-G*) regions, plus one nuclear locus (*ITS*) (S1 File), were used for subsequent phylogenetic analyses. The newly obtained sequences of *Cedrela* sp. from Tamaulipas and *C. odorata* were aligned along with GenBank sequences, assembling a final dataset that included 28 samples of *Cedrela* from Mexico, Central and South America and one sample of the sister genus *Toona*. Additionally, sequences from two Meliaceae taxa outside of Cedreleae were included as outgroup taxa to root the tree; *Azadirachta indica* and *Swietenia macrophylla*. Sequences for each genomic region were aligned using the MAFFT algorithm [38] with default parameters, and alignments for individual loci were concatenated into a single data matrix.

### Molecular phylogenetic analyses

A Bayesian phylogenetic analysis (BI) was performed on the full dataset of concatenated sequences using MrBayes 3.2.6 [39]. Two simultaneous runs (each with four chains) of the Metropolis-coupled Markov chain Monte Carlo (MCMC) were carried out for 5 million generations with a sampling frequency of parameters every 500^th^ generation. The best-fit models were selected independently for each partition according to the Akaike information criterion with jModelTest2 [40]. Stationarity and convergence of the Markov chains were assessed by checking the variation of log-likelihood values throughout the run generations with Tracer 1.7.2. [41]. The first 2500 sampled trees were excluded from the analysis as a burn-in; the remaining trees were used to generate a 50% majority rule consensus tree. Additionally, Maximum Likelihood (ML) analyses were performed with IQ-TREE 2.3.6. [42] using the -m TESTMERGE option to find the best-fit partitioning scheme by possibly merging partitions to reduce overparameterization. Support values for the inferred groups were estimated from 1000 ultrafast bootstrap replicates [43]. The tree from the BI analysis was used as the final figure, including support values for the nodes from both BI and ML analyses using the R library “ggtree” [44].

### Nomenclature

The electronic version of this article in Portable Document Format (PDF) in a work with an ISSN or ISBN will represent a published work according to the International Code of Nomenclature for algae, fungi, and plants, and hence the new names contained in the electronic publication of a PLOS ONE article are effectively published under that Code from the electronic edition alone, so there is no longer any need to provide printed copies.

In addition, new names contained in this work have been submitted to IPNI, from where they will be made available to the Global Names Index. The IPNI LSIDs can be resolved and the associated information viewed through any standard web browser by appending the LSID contained in this publication to the prefix http://ipni.org/. The online version of this work is archived and available from the following digital repositories PubMed Central, LOCKSS.

## Results

*Cedrela* sp. from Tamaulipas was first collected at Rancho El Cielo Biosphere Reserve in May 2018. A closer examination revealed substantial qualitative and quantitative morphological differences with other closely related species (Table 1). Additionally, anatomical data and phylogenetic analyses of chloroplast and nuclear DNA sequences, allowed us to conclude that we were dealing with an undescribed species of *Cedrela* from the northeastern Sierra Madre Oriental biogeographic province in México.

### Taxonomic treatment

**Fig 1:**
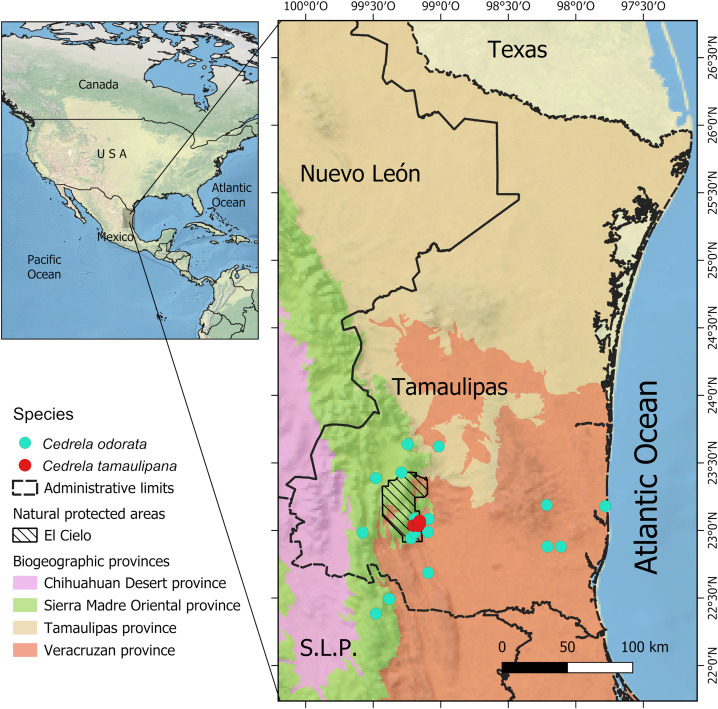
Distribution of *Cedrela* at the northernmost edge in the Atlantic slopes of Tamaulipas and San Luis Potosí, México. *Cedrela tamaulipana* (red circles) and *C. odorata* (turquoise circles). Source of data: field observations and GBIF [46]; Natural Earth [47] for administrative boundaries, shaded relief, and water. Biogeographical provinces were redrawn after Morrone [48]. The boundaries of El Cielo natural protected area are based on the official declaration by the state government of Tamaulipas [49]. Figure is similar but not identical to the original peer-reviewed image and is therefore for illustrative purposes only.

**Fig 2:**
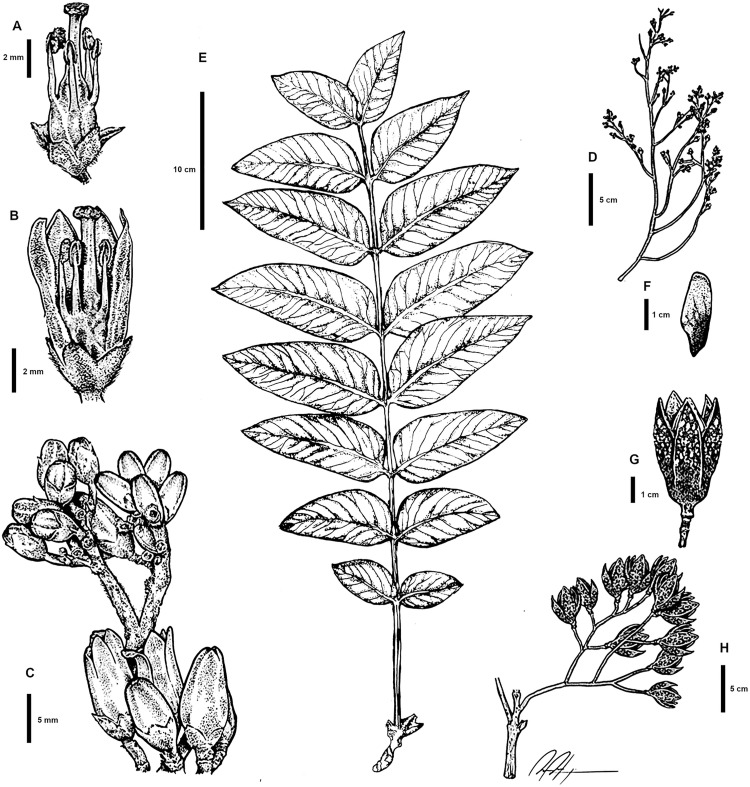
Vegetative and reproductive characters of *C. tamaulipana.* A–B. Dissected flowers. C. Cymule. D. branch with inflorescence during development, before anthesis. E. Leaf. F. Seed. G. Capsule. H. Infructescence. Illustration by Daniel Barba.

**Fig 3:**
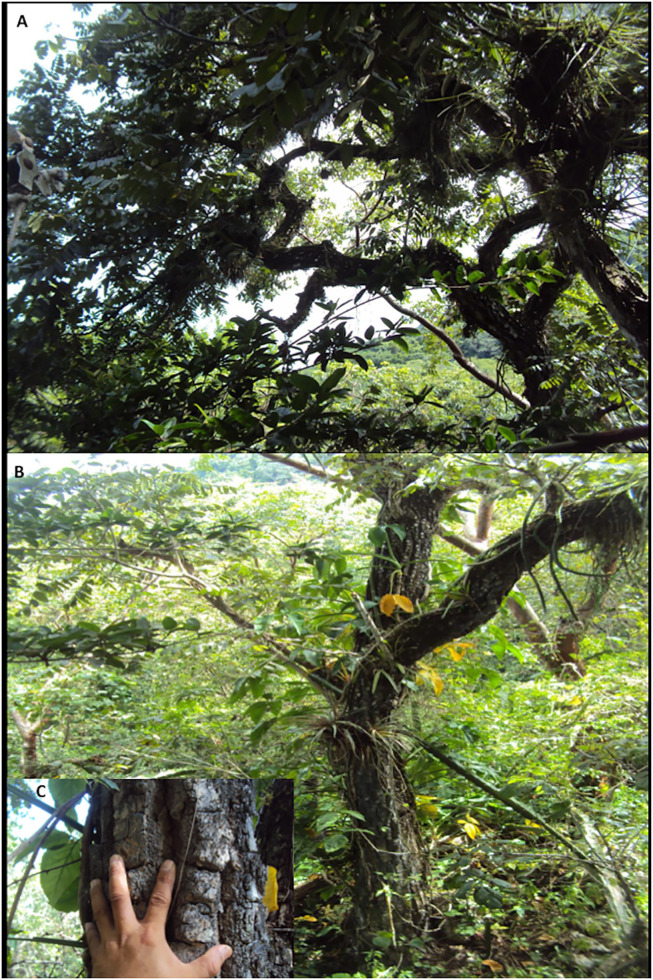
Habitat of *Cedrela tamaulipana* in Rancho El Cielo Biosphere Reserve, Tamaulipas, Mexico. A–B. Tree of *C. tamaulipana* with tortuous branching. C. Detail of its corky bark.

**Fig 4:**
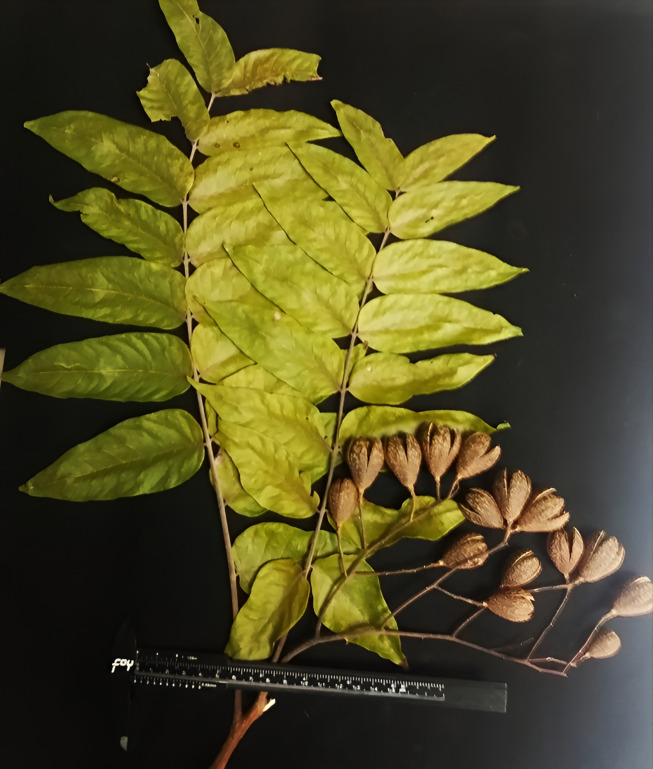
Specimen of *Cedrela tamaulipana.* Branch with leaves and capsules; type material.

**Fig 5:**
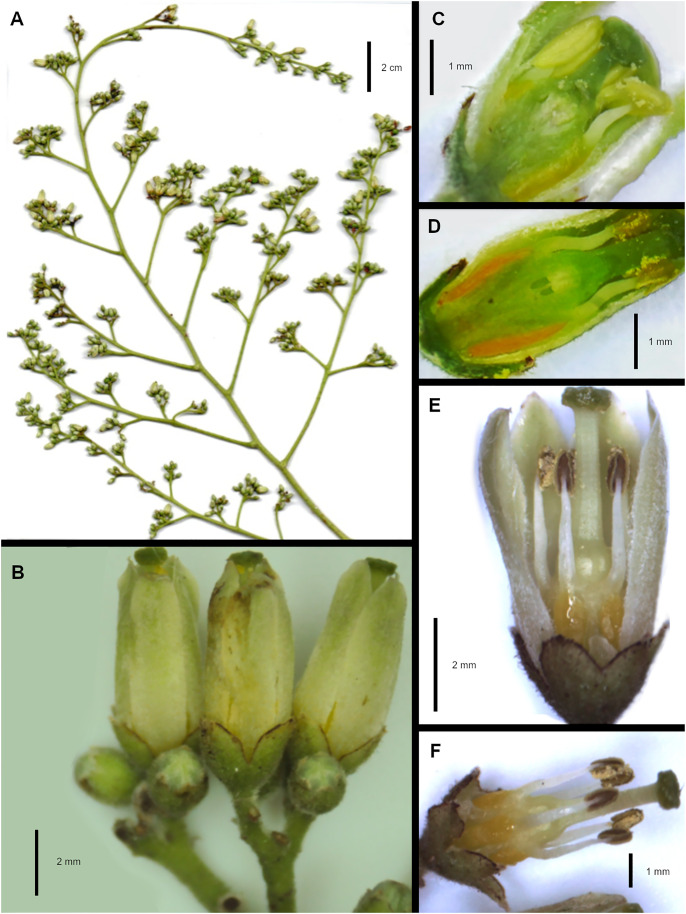
*Cedrela tamaulipana,* type material. A. Inflorescence. B. Flowers with barely exert stigmas. C. Dissected developing flower. D. Dissected nearly mature flower. E. Mature flower with sepals and petals; one petal removed. F. Mature flower with all five petals removed.

**Fig 6:**
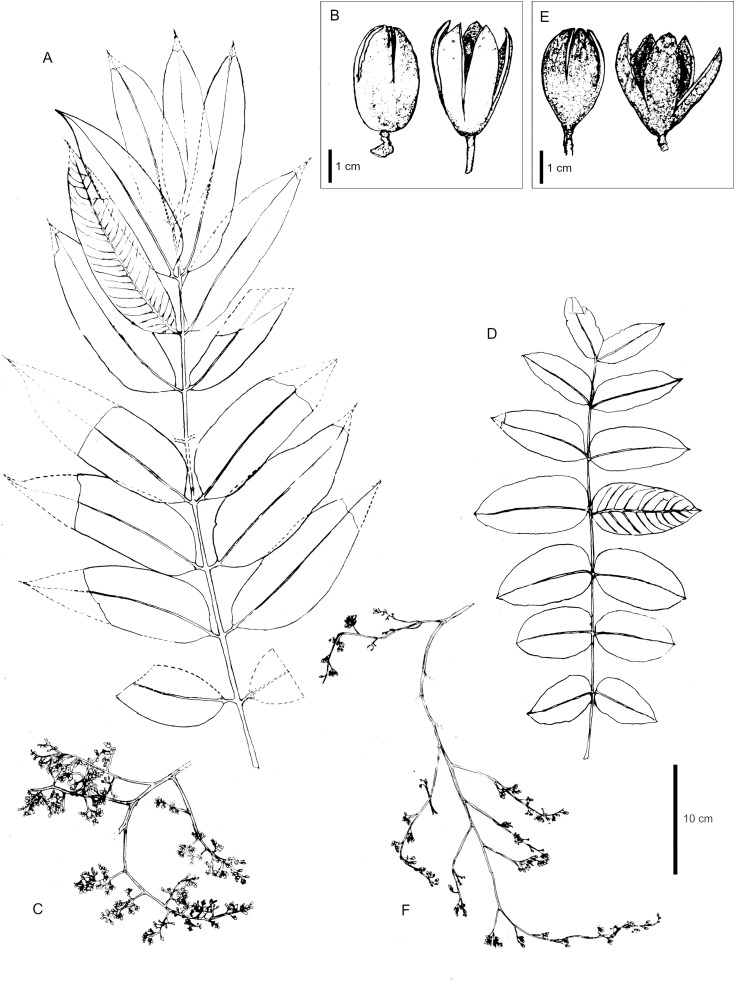
Comparative morphology of *Cedrela monroana* (A–C) vs. *C. tamaulipana* (D–F). A, D. Leaves. B, E. Ripen fruits, closed and open. C, F. Inflorescences. Source: A, C, *Martínez 265* (vouchers at B, LAGU); B, *Galan 6026* (voucher at LAGU), *Monro, Monterossa & Carballo 3789* (voucher at BM); D–F, *Berrones-Morales 1* (syntype material at IBUG). Scale bar of 10 cm applies to A, C–D, F and of 1 cm to B, F.

***Cedrela tamaulipana*** A.Vázquez & Gallardo-Yobal **sp. nov.** [urn:lsid:ipni.org:names:________] Type: MEXICO. Tamaulipas: Municipio de Gómez Farías, Alta Cima, 6.81 km al Noroeste de Gómez Farías y 1.08 km al Oeste de Alta Cima, on karstic topography with subdeciduous forest, 1042 m in elevation, 23°4′48.34″ N, 99°9′44.28″ W, 01 May 2018 (fl, fr), *M. Berrones-Morales 1* (Holotype: UAT [UAT-22868], isotype: IBUG [IBUG-214565]). — Figs 1–6.

#### Diagnosis.

*C*edrela tamaulipana** shares with *C. monroana* T.D. Penn. a similar leaf length (petiole + rachis), leaflet shape and glabrous leaf condition, number of leaflet pairs, leaflet apex, lax inflorescence, and fruit size. However, it differs from the latter in having a shorter height (up to 9.0–10.0 vs. up to 23.0 m); thinner young branches (4.0–5.0 vs. 10.0–15.0 mm); shorter space between leaflet pairs along the rachis (3.3–4.3 vs. 5.5–6.0 cm); shorter petiolules (1.0–2.0 vs. 3.0–9.0 mm); smaller leaflets (7.0 × 3.6–11.3 × 4.7 vs. 14.0 × 5.0–19.0 × 6.9 cm); smaller leaflet length-to-width ratio (1.7–2.4 vs. 2.7–2.8); less numerous secondary leaflet veins (9–11 vs. 15–19); shorter panicles (27.0–35.0 vs. 40.0–60.0 cm); petals green-yellowish vs. pale pinkish to deep reddish-purple; fruits broadly obovoid to pyriform vs. ellipsoid to slightly obovoid; mature capsules brown vs. purple; capsule with thicker valves (4.0–5.0 vs. 1.0–1.5 mm); dehiscing valves splitting at an angle of >20 degrees with prominent lenticels vs. barely splitting at an angle of <20 degrees, with inconspicuous lenticels.

#### Description.

Tree 9.0–10.0 m tall, 0.2–1.0 m DBH; young branches 4.0–5.0 mm in diameter, smooth, pale buff, with some elongate lenticels, glabrous. Leaves paripinnate, 40.0–60.0 cm long, glabrous; petioles 7.0–9.0 cm long, greenish in juvenile leaves, glabrous; leaflets 6–9 pairs, opposite, 7.0 × 3.6–11.3 × 4.7 cm, length-to-width ratio 1.7–2.4, elliptic to lanceolate, apex acute to acuminate, base asymmetric, glabrous, bright beam, glabrous in the middle nerves, sessile to subsessile; leaflet secondary veins 9–11; rachis 30.0–50.0 cm long; distance between leaflet pairs 3.3–4.3 cm; petiolules 1.0–2.0 mm. Panicles 27.0–35.0 cm long, lax, angle of primary branches ca. 45 degrees. Flowers 8.0–9.0 mm long, pentamers; 2.0–2.5 mm long; sepals united 0.5–1.0 mm, irregularly lobed; petals 7.0–8.0 mm long, most of their length at anthesis ½ or ⅓, fused to the androgynophore, oblong, green-yellowish, pubescent. The androgynophore is columnar, 3.0–4.0 × 1.0–1.2 mm, 5-sided; stamens 3.0–3.5 mm long, glabrous, with anthers 1 mm long; ovaries 1.1–1.3 mm long, penta-lobed; styles 1.5–2.0 mm long; stigmas capitate, 1.0–1.3 mm wide. Fruits 3.0–3.5 × 1.5–1.8 cm, broadly obovoid to pyriform; central column 7.0–9.0 mm in diameter, with sharp or truncated apex, pericarps 4.0–5.0 mm thick with few prominent ocher lenticels regularly distributed throughout the upper half distal surface, capsule valves 4.0–5.0 mm thick, curved inward, brown, with prominent lenticels. Seeds 2.5–2.7 cm long, wings 1.5–1.8 cm, non-translucent and reddish-brown.

#### Distribution, habitat and phenology.

*Cedrela tamaulipana* is only known from El Cielo Biosphere Reserve, Municipality of Gómez Farías, Tamaulipas, Mexico. It occurs along an elevational gradient (300–1100 m) from tropical subdeciduous forest to oak-forest with cloud forest elements. Flowering occurs from May to June and fruiting from July to August.

#### Eponymy and ethnobotany.

**Fig 7:**
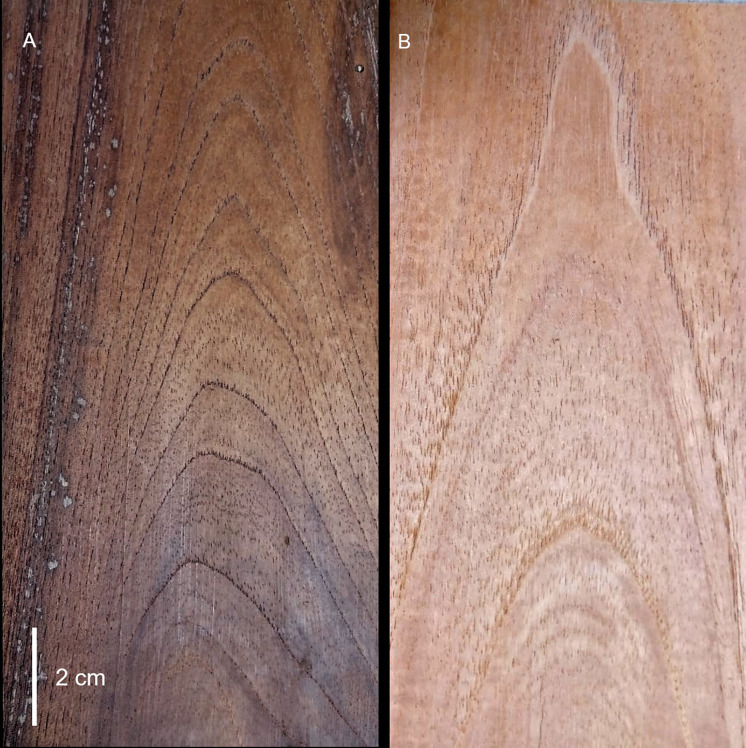
Tangential section of wood from *Cedrela tamaulipana* and *C. odorata*. A. *C. tamaulipana*. B. *C. odorata.* Photographs by Sergio Ignacio Gallardo-Yobal.

The species is named after the northeastern Mexican state of Tamaulipas, where the type locality is located at El Cielo Biosphere Reserve. *C. tamaulipana* is locally known as “cedro negro” (black cedar) or “cedro nogal” (walnut cedar) due to its dark wood color (Fig 7), peculiar tree structure and shape of its leaves, which resemble a walnut.

Ecologically, *Cedrela tamaulipana* thrives at higher latitudes than *C. monroana*, 23°00′–23°10′N vs. 13°35′–13°40′ and its flowering phenology occurs from May to June vs. from August to October. Additionally*, Cedrela tamaulipana* differs from *Cedrela saxatilis*, in having shorter petiolules 1.0–2.0 vs. 2.5–5.0 mm, leaflets elliptic to lanceolate vs. broadly lanceolate; fewer secondary veins (9–11 vs. 15–16), more inclined angle, in degrees, of panicle branches (45 and upward vs. 30–40 and downward); flowers green-yellowish vs. purplish; and longer fruits 3.0–3.5 vs. 2.0 cm. Ecologically, *Cedrela tamaulipana* thrives at higher latitudes than *C. saxatilis* (23°00’–23°10’N vs. ca. 16°–19° N), lower elevations (300–1100 vs. 1500–2100 m) and its flowering is from May to June vs. September.

*Additional specimens examined (paratypes)*.—MEXICO. Tamaulipas: Municipality of Gómez Farias, 5 km NE 350 m in elevation, 1 May 2019, *Gallardo-Yobal 145* (UAT-22998!); same location, 8 November 2022 *Berrones-Morales 26* (IBUG- 217371), *Berrones-Morales 27* (IBUG-217372).

### Proposal to rehabilitate the name *Cedrela saxatilis*

**Fig 8:**
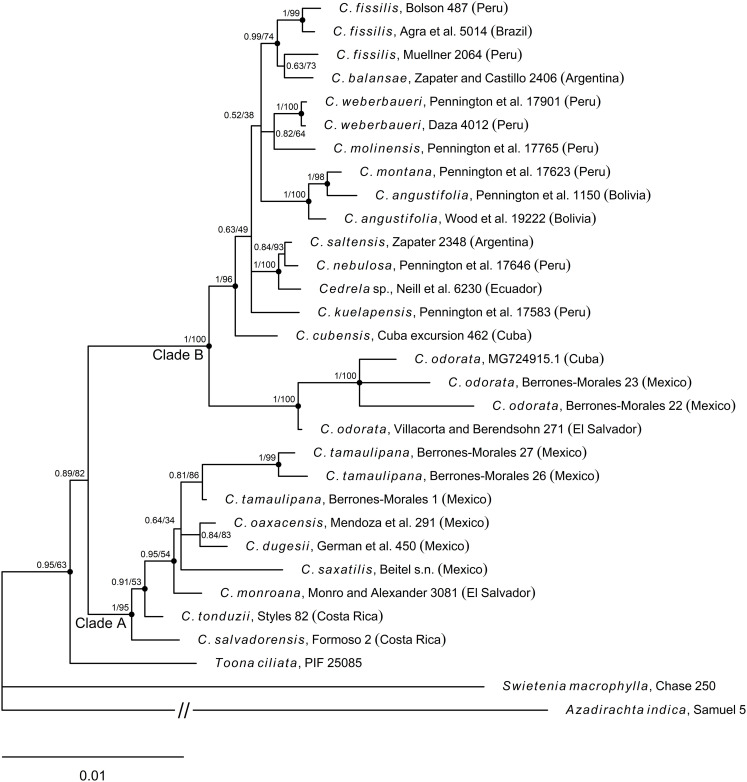
Majority rule (50%) consensus tree from the phylogenetic analysis with MrBayes inferred from DNA sequences of 10 genomic regions; nine from the chloroplast (*accD*, *matK*, *rbcL*, *trnH*-*psbA*, *psbB*-*T*-*N*, *rpl16*, *rpoB*, *rpoC1*, *trnS*-*G*) and one nuclear (*ITS*). Nodes are annotated with posterior probability (left) and bootstrap support values (right). Dots indicate well-supported nodes from BI (PP > 0.9). The scale bar represents the number of nucleotide substitutions per site. The letter “C.” in taxon names is an abbreviation for *Cedrela*.

*Cedrela saxatilis* has consistently been treated as a synonym of *C. oaxacensis* [7,50]. Here we propose the validity of *C. saxatilis* as a good species in its own merit, based on morphological and phylogenetic differences between these species; *C. saxatilis* differs from *C. oaxacensis* in: (1) having wider leaflets 5.0–6.0 vs. 3.0–4.5 cm; (2) higher leaflet length to width ratio 2.2–2.3 vs. 1.7–2.1; (3) very slightly pubescent leaves vs. abaxially pale tomentose or villose; (4) shorter capsules 2.0 vs. 3.5–4.0 cm; (5) pendulous capsules vs. erect; and (6) lenticels on capsule valves absent vs. present. We found that the sample *Beitel s.n.* (OSC) included in our study, morphologically matched the type material of *C. saxatili*s, *Rose & Painter 6950* (US) (Table 1). Furthermore, in our inferred phylogeny, this sample was not recovered within the clade including *C. oaxacensis* and *C. dugesii* (Fig 8)*.*

### Key to the Mexican species of *Cedrela* and the closely related *C. monroana*

1a Capsules ≥ 5 cm long … 2
  2a Trees up to 15 m, flowering leafless; inflorescence lateral on old wood, leaves including petiole 40.0–70.0 cm long; leaflet length to width ratio 2.5–2.8; secondary veins 15–20, panicle branches at 30–45 degrees, capsules 8.0–14.0 cm (W, central and SE Mexico to Costa Rica) … *C. salvadorensis*
  2b Trees up to 50 m, flowering when in leaf; inflorescence terminal; leaves including petiole 15.0–25.0 cm long; leaflet length to width ratio 1.8–1.9; secondary veins 5–11, panicle branches at ≈90 degrees, capsules 5.0–8.0 cm (S Mexico to W Panama) … *C. tonduzii*
1b Capsules < 5 cm long … 3
  3a Lower leaflet surface pubescent to tomentose or with minute appressed hairs in the interstice of veins … 4
    4a Leaflets discolorous, abaxially appressed puberulous, domatia absent, flowers in dense clusters (NW Mexico) … *C. discolor*
    4b Leaflets not discolorous, abaxially pubescent to tomentose, domatia hair-filled, flowers in lax clusters (S. Mexico) … *C. oaxacensis*
  3b Lower leaflet surface essentially glabrous or rarely puberulous … 5
    5a Domatia present … 6
      6a Trees 10–15 m; leaflet length to width ratio 2.7–2.8, leaflets ovate to ovate-triangular and apex long acuminate, capsules with inconspicuous lenticels (central Mexico) … *C. dugesii*
      6b Trees 20–40 m; leaflet length to width ratio 3.2–3.3, leaflets lanceolate to oblong-lanceolate and apex short acuminate, capsules with evident white lenticels (Mexico, Central America and the Caribbean) … *C. odorata*
    5b Domatia absent … 7
      7a Petiolules 1–2 mm, secondary veins 9–11, leaflets elliptic to lanceolate, flowers green-yellowish (NE Mexico) … *C. tamaulipana*
      7b Petiolules 2.5–9 mm, secondary veins 15–19, leaflets broadly lanceolate, flowers pinkish to reddish-purple … 8
        8a Trees up to 23 m, bark fissured, obscure grayish-brown, leaves including petiole 45.0–55.0 cm long, panicles 40.0–60.0 cm long, panicle branches at ≈45–90 degrees, capsules 3.5–4.0 cm long (Guatemala, El Salvador) … *C*. *monroana*
        8b Trees 4–7 m, bark smooth and reddish, leaves including petiole 20–40.0 cm long, panicles ≥30 cm long, panicle branches reflexed at 30–40 degrees, capsules 2.0 cm long (S Mexico) … *C. saxatilis*

### Wood anatomy

**Fig 9:**
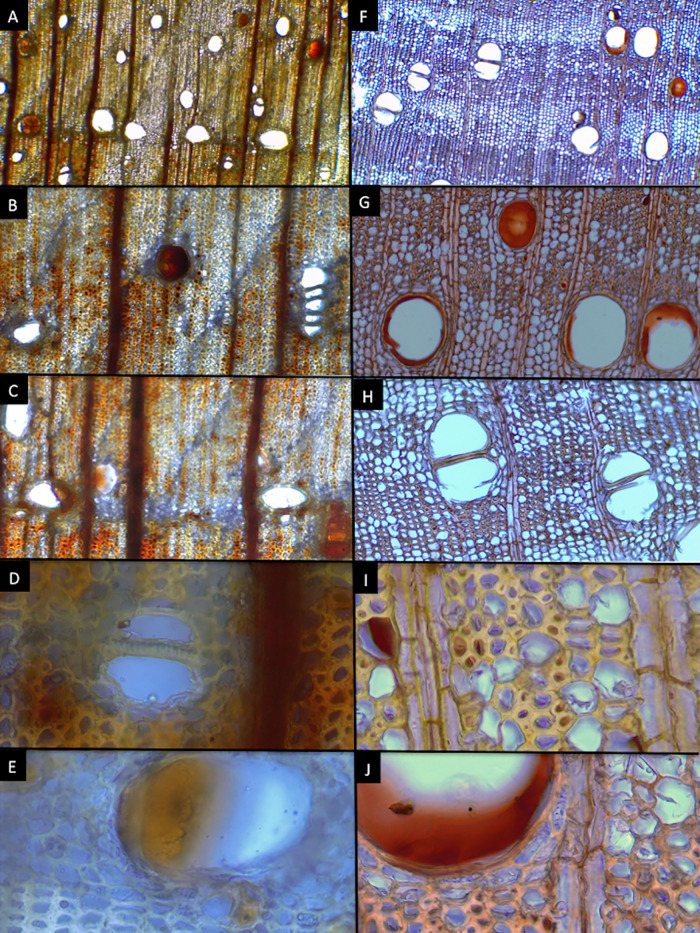
Transverse section of histological samples of wood from *Cedrela tamaulipana* and *C. odorata.* *Cedrela tamaulipana* (A–E) vs. *C. odorata* (F–J). A–E. Magnification used for images of the first row was 4 × , for the 2nd and 3rd rows 10× and for the 4th and 5th rows 40 × . Photographs by Hilda Palacios.

**Fig 10:**
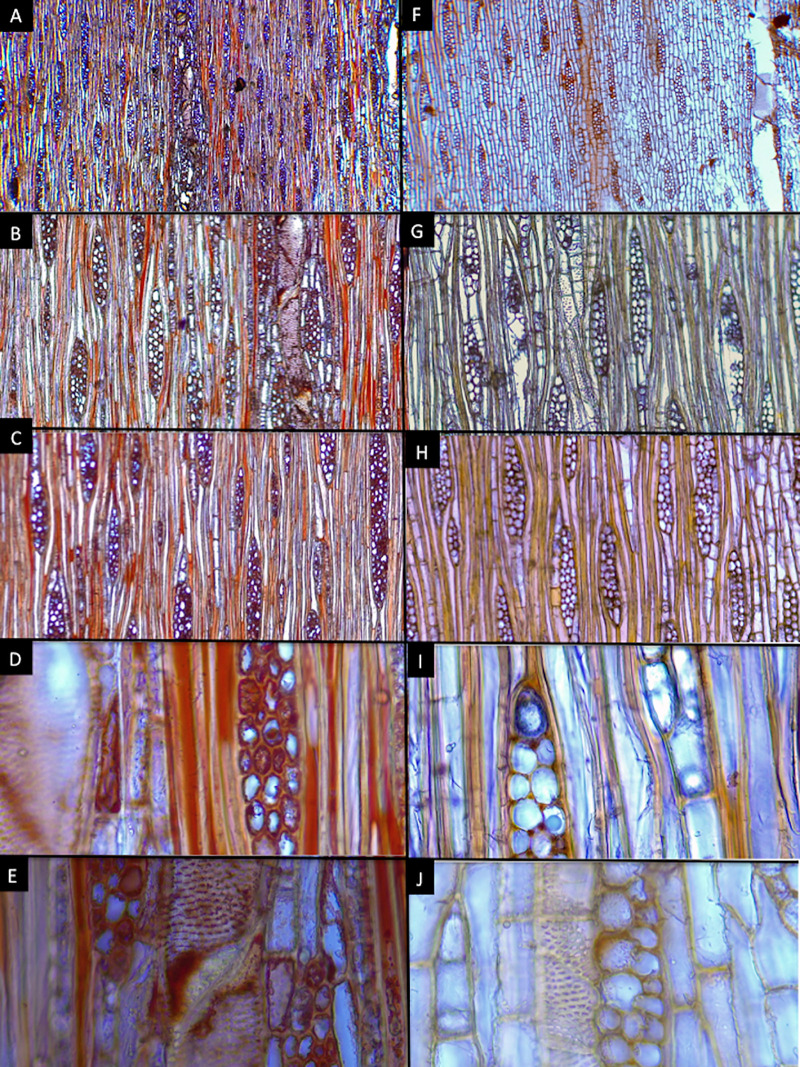
Tangential section of histological samples of wood from *Cedrela tamaulipana* and *C. odorata.* *Cedrela tamaulipana* (A–E) vs. *C. odorata* (F–J). Magnification used for images of the first row was 4 × , for the 2nd and 3rd rows 10× and for the 4th and 5th rows 40 × . Photographs by Hilda Palacios.

*Cedrela tamaulipana* has distinct growth rings, marked by vessel diameter differences and marginal parenchyma (Fig 9A, C, E). Wood is semi-ring-porous; the tangential diameter of earlywood vessels is 168.20 µm and latewood 59.33 µm, arranged as solitary or in radial groups of 2–3 and circular in outline (Fig 9B, D); perforate plates are simple and inter vessel pits alternate, polygonal (Fig 10E). Fibers are non-septate with minutely simple pits, diameter 17.66 µm, and 3.32 µm in wall thickness. Apotracheal parenchyma is diffuse, paratracheal scanty or vasicentric, and marginal have narrow bands up to 4 cells wide, strands commonly of 4–6 cells (Fig 10C). Uniseriate rays are rare, multiseriate rays are 2–4-seriate, most common 3-seriate (Fig 10A–D), 340.29 µm high and 45.56 µm wide, with procumbent cells and marginal cells upright or sometimes squared. Dark-staining deposits are present in several vessel lumina, most fibers and ray cells (Figs 9, 10). Prismatic crystals are deposited in non-chambered axial parenchyma cells and rays (Fig 10B).

### Molecular phylogenetic analysis

The final matrix of concatenated sequences from 10 genomic regions comprised 7072 aligned base pairs, of which 570 were variable sites, and 199 phylogenetically informative. The nucleotide substitution models that best fit each DNA partition, and the summary statistics of DNA sequences, are presented in Table 2.

**Table 2:** Summary statistics for the 10 genomic regions used in phylogenetic analyses of *Cedrela* and other representatives within Meliaceae.

	*accD*	*matK*	*psbA-trnH*	*psbB-T-N*	*rbcLa*	*rpl16*	*rpoB*	*rpoC1*	*trnS-G*	*ITS*
Aligned base pairs	249	988	485	785	714	939	623	844	532	913
Variable sites	2	96	30	62	29	29	8	100	6	208
Parsimony informative sites	2	13	21	30	15	9	7	22	6	74
Model of nucleotide substitution	HKY	GTR	GTR + G	GTR + G + I	F81	GTR	HKY	GTR + I	GTR + G + I	GTR + G

Both BI and ML phylogenetic analyses, essentially recovered the same tree topology, except for different support values for some groups. *Cedrela* splits into two well-supported clades; clade A (PP = 1.0, BS = 95%) which includes species from Mexico, Costa Rica and El Salvador, and clade B (PP = 1.0, BS = 100%) including samples from Cuba, Mexico and the rest of the Central and South American species (Fig 8). Both BI and ML analyses confirm the monophyletic nature of *Cedrela* (PP = 0.89, BS = 82%) with *Toona* recovered as a sister lineage.

The species described here as *C. tamaulipana* sp. nov. is placed within clade A, as closely related to *C. dugesii*, *C. monroana*, *C. oaxacensis*, and *C. saxatilis* (voucher *Beitel* s.n.). Although the relationships among these species were not fully resolved, *C. tamaulipana* appears as a genetically distinct group (PP = 0.81, BS = 86%) separated from the other species (Fig 8). *Cedrela salvadorensis* and *C. tonduzii* are recovered as more divergent taxa within this clade A.

Within clade B, *Cedrela odorata* s. str. (voucher *Villacorta and Berendsohn 271* from El Salvador) is placed as a sister taxon to *C*. *odorata* from Tamaulipas, Mexico (vouchers *Berrones-Morales 22* and *23*) and Cuba (voucher Thuenen-ID CEODO_205_2, GenBank accession MG724915.1); all these samples were recovered as a more divergent and well-supported (PP = 1.0, BS = 100%) monophyletic group within clade B. The South American species (*C. angustigolia* DC., *C. balansae* C. DC., *C. fissilis* Vell., *C. kuelapensis* T.D. Penn. & Daza, *C. molinensis* T.D. Penn. & Reynel, *C. montana* Moritz ex Turcz., *C. nebulosa* T.D. Penn. & Daza, *C. saltensis* M.A. Zapater & del Castillo, *C. weberbaueri* Harms and *Cedrela* sp. voucher *Neill et al. 6230*) and one species from the Antilles (*C. cubensis* Bisse) (voucher *Cuba excursion 462*, S1 File), were recovered as a well-supported monophyletic group (PP = 1.0, BS = 96%). The relationships among South American species were poorly resolved and the results from BI and ML analyses were slightly different, with low support values for the internal nodes.

## Discussion

Evidence from an integrative approach, involving morphological, ecological and molecular data, support the recognition of *Cedrela tamaulipana* as a new species. Likewise, based on morphological and phylogenetic evidence we propose the validity of *C. saxatilis* as a distinct species.

### Morphology

*Cedrela* *tamaulipana* has a tortuous trunk, with branches in a zigzag pattern, unlike most species of *Cedrela* [7]. It shares the short habit (<15 m tall) of *C*. *dugesii* and *C*. *oaxacensis*, however, its bark is blackish, differing from those of *C*. *dugesii* (gray) and *C*. *oaxacensis* (reddish gray). It also differs from *C. oaxacensis* in having a corky and deeply fissured bark, distinct wood growth rings, panicle branches at an angle of ca. 45 degrees, flowers green-yellowish, fruit broadly obovoid to pyriform and thicker, strongly curved carpel valves [51,52].

*Cedrela saxatilis* has been treated as a synonym of *C. oaxacensis* [7,50], however, based on the morphological similarities found between the sample initially labeled in our study as *C.* aff. *odorata* NYBG accession 683/89 and the type material of *C. saxatilis,* along with our phylogenetic results, we agree with Joseph Nelson Rose that *C. saxatilis* is a distinct species*.* When naming the latter, Rose [53] had a relatively good understanding of the taxonomy of Mexican *Cedrela* species and rightfully contrasted *C. saxatilis* against the other glabrous species, and not against the abaxially pubescent *C. oaxacensis*. It is also noteworthy to mention that Finch et al. [54], in their phylogenetic analysis using whole chloroplast genomes, found that such a sample (labeled in their study as *C. odorata* CEOD-NYBG) represents a more divergent taxon, sister to the other *Cedrela* species included in their analysis. We have also recovered it as a divergent taxon, not grouped with any other closely related species within clade A (Fig 8); particularly, it was not recovered within the clade including *C. oaxacensis* + *C. dugesii*.

### Wood anatomy

The new species shares most wood characteristics with the other *Cedrela* species but seems to be unique in the number of dark-staining deposits in fiber lumina as well as in parenchyma and rays (Figs 9, 10). *C. tamaulipana*, like most species of *Cedrela*, has ring or semiring porous wood; other species like *C. montana* have diffuse-porous wood. *Cedrela tamaulipana* differs from *C*. *odorata* in having narrower earlywood vessel diameter (168.20 vs. 255.21 µm), narrower latewood vessel diameter (59.33 vs. 120.52 µm), narrower fiber diameter (17.66 vs. 26.22 µm) with thinner walls (3.32 vs. 4.59 µm). Although strong cell size differences for vessels and fibers were detected between *C. tamaulipana* and *C*. *odorata,* comparisons with other species are needed; because quantitative data are difficult to obtain from references, especially for earlywood and latewood vessel diameter and fiber wall thickness.

Additionally, the prismatic crystals in non-chambered cells are shared with *C. odorata* and *C. montana* and different in the chambered ones compared to *C. salvadorensis* and *C. tubiflora*; these crystals are lacking in *C. balansae*. *C. tamaulipana* showed 1–3-seriate rays, similar to *C. balansae*, *C. odorata*, *C. salvadorensis* and *C. tubiflora* [25], but wider 2–6 seriate rays are found in *C. montana* [55].

### Molecular phylogenetic analysis

Results from phylogenetic analyses confirm that *C*. *tamaulipana* represents a genetically distinct taxon that belongs to the clade of Mexican and Central American species of *Cedrela* (Fig 8, clade A). Similar to the results of Muellner et al. [6] and Koecke et al. [5], our phylogeny shows that the genus comprises two monophyletic groups with distinct geographical affinity. Clade B mainly includes species from South America, with some early-diverged lineages from Central America and the Antilles. In contrast, clade A includes species from Mexico and Central America, where *C. tamaulipana* is placed. Interestingly, despite the sympatry of *C. odorata* and *C. tamaulipana*, the latter is recovered within clade A as a monophyletic group (PP = 0.81, BS = 86), reflecting its phylogenetic distinctiveness (Fig 8). Hence, besides the morphological and ecological differences, the molecular evidence also supports that *C. tamaulipana* represents a distinct taxon.

The analyzed sequence data could mostly resolve the relationships among members of clade B, although with low support for some of them. Similarly, Koecke et al. [5], using a matrix of concatenated sequences from nuclear and cpDNA, obtained a lack of support for several relationships among members of this clade (clade II in their study). For instance, they recover *C. odorata* s. str. as a divergent lineage sister to samples from Central and South America, all included in a recently evolved clade but with a very low support value (PP = 0.58). Pennington & Muellner [7], combining nuclear and plastid data, recovered *C. odorata* s. str. as a sister lineage to samples only from South America but also with a very low support value (PP = 0.44). Similarly, Muellner et al. [6] recovered *C. odorata* s. str. from Central America as a sister group to all Southern American species, except *C. angustifolia* and *C. montana*, which formed a distinct and more basal clade. We recovered *C. odorata* from Tamaulipas, Cuba, and El Salvador as an early diverged clade (PP = 1.0, BS = 100%) within clade B (PP = 1.0, BS = 100%), sister to a clade including all sampled South American species, plus *C. cubensis* from Cuba (Fig 8). Our result is compatible with that of Finch et al. [54], who used whole chloroplast genome sequences from *Cedrela* and closely related genera. The five species of *Cedrela* included in their study were recovered as a monophyletic group, which were also recovered as monophyletic in our study within clade B, except for their sample labeled as *C. odorata* CEOD-NYBG, which corresponds to *C. saxatilis Beitel s.n.* in our study, placed within clade A (Fig 8). Similarly, Finch et al. [54] recovered this sample as a more divergent taxon, sister to all of the *Cedrela* species they included.

One possible cryptic species is represented by the sample of *Cedrela* sp. from Ecuador (*Neill* et al. *6230*); even though this specimen was repeatedly determined as *C. odorata*, by T. D. Pennington, our phylogenetic analyses place it with high support values (PP = 1.0, BS = 100%) as a sister taxon to *C. saltensis* and *C. nebulosa*, from Argentina and Peru, respectively. This relationship was also found by Muellner et al. [6]; hence this taxon requires a closer taxonomic revision.

Within clade A, the earliest divergent species was *C. salvadorensis*, in accordance with the results obtained by Muellner et al. [6] and Pennington & Muellner [7]. The close relationship between *C. dugesii* and *C. oaxacensis* (PP = 0.84, BS = 83%) is also in agreement with the results of Muellner et al. [6], Pennington & Muellner [7], and Cavers et al. [3]. However, Koecke et al. [5] recovered *C. dugesii* as a sister taxon to *C. monroana*, and *C. oaxacensis* as a sister taxon to *C. salvadorensis*, although the latter relationship with very low support (PP = 0.6). In contrast, we recovered *C. monroana* as an early divergent taxon within the five-species subclade (PP = 0.95, BS = 54%) including *C. dugesii*, *C. oaxacensis*, *C. tamaulipana* and *Cedrela saxatilis* (Fig 8, clade A). Contrary to previous studies [6,7], we recovered *C. tonduzii* in a basal position within clade A, with a relatively high posterior probability for the group (PP = 0.91) but with a contrasting low bootstrap value (BS = 53%).

Interestingly, we found that *C. tamaulipana trnH-psbA* sequence, corresponds to the haplotype H21 reported by Koecke [56] for *C. dugesii* (*German et al. 450*) and *C. monroana* (*Monroe & Alexander 308*). Likewise, there is only one nucleotide difference between the haplotype H21 and the haplotype for the same locus from the sample of *C. saxatilis* from Oaxaca (*Beitel* s.n.). According to Koecke [56], this haplotype is restricted to Mexico and Central America, thus suggesting a shared evolutionary history for these taxa, also supported by our phylogenetic results which recovered clade A as monophyletic.

### Ecology and distribution pattern

Species of *Cedrela* from Mexico and Central America recovered in clade A occur from 8°00’N to 22°14’N of latitude [7], except *C. tamaulipana*, which has the most septentrional distribution; between 23°00’N and 23°10’N (Table 1). *Cedrela tamaulipana* is confined to the Sierra Madre Oriental, and thus, it is latitudinally isolated from all other species in clade A, including the geographically close species *C. duguesii*, which occurs in the Chihuahuan Desert, southern Sierra Madre Occidental and the Trans-Mexican Volcanic Belt. Likewise, *C. oaxacensis* is only found towards the south; in the Balsas Basin, Sierra Madre del Sur and the Pacific Lowlands. On the other hand, the altitudinal range of *C. tamaulipana* overlaps with some of the Mexican *Cedrela* species, except with *C. duguesii*, *C. saxatilis*, and *C. discolor* (Table 1) [7]. Southern *Cedrela* species tend to have a broader altitudinal niche breadth and higher elevations. The habitat of *C. tamaulipana* (subdeciduous forest), differs from the habitat reported for most of its sister species from clade A, except for the Central American *C. monroana,* which thrives in a similar habitat. *C. duguesii* and *C. salvadorensis* grow in tropical deciduous forests, while *C. oaxacensis* and *C. tonduzii* occur mostly in pine-oak forests and the latter occasionally in cloud forests along with species of *Liquidambar* and *Podocarpus* [7].

## Conclusions

*Cedrela tamaulipana* is a distinct species based on morphological, molecular and ecological evidence; hence, *Cedrela*´s diversity, including the here reinstated *C. saxatilis*, is now updated to 23 species. Within the monophyletic *Cedrela*, *C. tamaulipana* belongs to a clade including taxa from Mexico and Central America. Based on morphological and phylogenetic evidence, *C. saxatilis* represents a distinct taxon, not a synonym of *C. oaxacensis*. Further taxonomic studies using an integrative approach are required to reveal the expected cryptic diversity of *Cedrela* in the Neotropics.

## Supporting information

S1 File Appendix 1. GenBank accession numbers, voucher information and geographic origin of Meliaceae samples used in phylogenetic analysis. (DOCX)

S2 File Appendix 2. Selected specimens (Exsiccatae) of Mesoamerican *Cedrela.* (DOCX)

## References

[1] Pennington TD, Styles BT. A generic monograph of the Meliaceae. Blumea. 1975;22:419–540.

[2] Cavers S, Navarro C, Lowe AJ. Chloroplast DNA phylogeography reveals colonization history of a Neotropical tree, Cedrela odorata L., in Mesoamerica. Mol Ecol. 2003;12(6):1451–60. doi: 10.1046/j.1365-294x.2003.01810.x 12755874

[3] Cavers S, Telford A, Arenal Cruz F, Pérez Castañeda AJ, Valencia R, Navarro C, et al. Cryptic species and phylogeographical structure in the tree Cedrela odorata L. throughout the Neotropics. Journal of Biogeography. 2013;40(4):732–46. doi: 10.1111/jbi.12086

[4] Heads M. Biogeography and ecology in a pantropical family, the Meliaceae. GBS. 2019;71(suppl.2):335–461. doi: 10.26492/gbs71(suppl.2).2019-22

[5] Koecke AV, Muellner‐Riehl AN, Pennington TD, Schorr G, Schnitzler J. Niche evolution through time and across continents: The story of Neotropical Cedrela (Meliaceae). American J of Botany. 2013;100(9):1800–10. doi: 10.3732/ajb.1300059 24018859

[6] Muellner AN, Pennington TD, Koecke AV, Renner SS. Biogeography of cedrela (meliaceae, sapindales) in central and South america. Am J Bot. 2010;97(3):511–8. doi: 10.3732/ajb.0900229 21622412

[7] Pennington TD, Muellner AN. A monograph of Cedrela (Meliaceae). Milborne Port, England: Dh Books. 2010.

[8] Palacios WA, Santiana J, Iglesias J. A new species of Cedrela (Meliaceae) from the eastern flanks of Ecuador. Phytotaxa. 2019;393(1):84. doi: 10.11646/phytotaxa.393.1.8

[9] Hágsater E, Duarte Salinas J, Jiménez machorro R, Pío-León JF, Millán Otero MG. Epidendrum petacaense, a new species of Orchidaceae from Sinaloa, Mexico. Phytotaxa. 2023;592(2):81–7. doi: 10.11646/phytotaxa.592.2.1

[10] Villanueva-Tamayo B, Morales-Puentes ME, Cruz OM, Aymard-Corredor GA. A New Species of Cedrela (Meliaceae) from a Colombian Dry Forest and an Updated Key for the Species of the Genus. Harvard Papers in Botany. 2023;28(2). doi: 10.3100/hpib.v28iss2.2023.n11

[11] Edmonds JM. The potential value of Toona species (Meliaceae) as multipurpose and plantation trees in Southeast Asia. Commonw For Rev. 1993;72:181–6.

[12] Mabberley DJ, Pannell CM, Sing AM. Flora Malesiana ser. I Meliaceae. Leiden, Netherlands: Rijksherbarium, Foundation Flora. 1995.

[13] Muellner AN, Pennington TD, Chase MW. Molecular phylogenetics of Neotropical Cedreleae (mahogany family, Meliaceae) based on nuclear and plastid DNA sequences reveal multiple origins of “Cedrela odorata”. Mol Phylogenet Evol. 2009;52(2):461–9. doi: 10.1016/j.ympev.2009.03.025 19348956

[14] Muellner AN, Samuel R, Johnson SA, Cheek M, Pennington TD, Chase MW. Molecular phylogenetics of Meliaceae (Sapindales) based on nuclear and plastid DNA sequences. Am J Bot. 2003;90(3):471–80. doi: 10.3732/ajb.90.3.471 21659140

[15] Muellner AN, Savolainen V, Samuel R, Chase MW. The mahogany family “out-of-Africa”: divergence time estimation, global biogeographic patterns inferred from plastid rbcL DNA sequences, extant, and fossil distribution of diversity. Mol Phylogenet Evol. 2006;40(1):236–50. doi: 10.1016/j.ympev.2006.03.001 16624592

[16] Muellner-Riehl AN, Rojas-Andrés BM. Biogeography of Neotropical Meliaceae: geological connections, fossil and molecular evidence revisited. Braz J Bot. 2022;45(1):527–43. doi: 10.1007/s40415-021-00770-4

[17] Cavers S, Navarro C, Lowe AJ. A combination of molecular markers identifies evolutionarily significant units in Cedrela odorata L. (Meliaceae) in Costa Rica. Conservation Genetics. 2003;4(5):571–80. doi: 10.1023/a:1025692321860

[18] Köcke AV, Muellner-Riehl AN, Cáceres O, Pennington TD. *Cedrela ngobe* (Meliaceae), a new species from panama and costa rica. Edinburgh J Bot. 2015;72(2):225–33. doi: 10.1017/s0960428615000098

[19] Finch KN, Jones FA, Cronn RC. Cryptic species diversity in a widespread neotropical tree genus: The case ofCedrela odorata. American J of Botany. 2022;109(10):1622–40. doi: 10.1002/ajb2.16064 PMC9827871 36098061

[20] Styles BT. Swietenioideae. In: Pennington TD, Styles T, Taylor DAH. Flora Neotropica Monograph. New York: New York Botanical Garden. 1981. 359–418.

[21] Villaseñor JL. Checklist of the native vascular plants of México. RevMexBiodiv. 2016;87(3). doi: 10.1016/j.rmb.2016.06.017

[22] Navarro C, Ward S, Hernández M. The tree Cedrela odorata (Meliaceae): a morphologically subdivided species in Costa Rica. Rev Biol Trop. 2002;50(1):21–9. 12298247

[23] Thiers B. The World’s Herbaria 2017: A Summary Report Based on Data from Index Herbariorum. New York: New York Botanical Garden. 2018.

[24] Radford AE, Dickison WC, Massey JR, Bell CR. Vascular plant systematics. New York: Harper & Row. 1974.

[25] IAWA Committee. Iawa list of microscopic features for hardwood identification. IAWA J. 1989;10:219–332.

[26] Wheeler EA. Inside Wood – A Web resource for hardwood anatomy. IAWA J. 2011;32(2):199–211. doi: 10.1163/22941932-90000051

[27] Aljanabi SM, Martinez I. Universal and rapid salt-extraction of high quality genomic DNA for PCR-based techniques. Nucleic Acids Res. 1997;25(22):4692–3. doi: 10.1093/nar/25.22.4692 9358185 PMC147078

[28] Muellner AN, Samuel R, Chase MW, Pannell CM, Greger H. Aglaia (Meliaceae): an evaluation of taxonomic concepts based on DNA data and secondary metabolites. Am J Bot. 2005;92(3):534–43. doi: 10.3732/ajb.92.3.534 21652432

[29] Levin RA, Wagner WL, Hoch PC, Nepokroeff M, Pires JC, Zimmer EA, et al. Family-level relationships of Onagraceae based on chloroplast rbcL and ndhF data. Am J Bot. 2003;90(1):107–15. doi: 10.3732/ajb.90.1.107 21659085

[30] Kress WJ, Erickson DL. A two-locus global DNA barcode for land plants: the coding *rbcL* gene complements the non-coding trnH-psbA spacer region. PLoS One. 2007;2(6):e508. doi: 10.1371/journal.pone.0000508 17551588 PMC1876818

[31] Sang T, Crawford DJ, Stuessy TF. Chloroplast DNA phylogeny, reticulate evolution, and biogeography of Paeonia (Paeoniaceae). American J of Botany. 1997;84(8):1120–36. doi: 10.2307/2446155 21708667

[32] Tate JA, Simpson BB. Paraphyly of Tarasa (Malvaceae) and diverse origins of the polyploid species. Syst Bot. 2003;28:723–37.

[33] Ford CS, Ayres KL, Toomey N, Haider N, Van Alphen Stahl J, Kelly LJ, et al. Selection of candidate coding DNA barcoding regions for use on land plants. Botanical Journal of the Linnean Society. 2009;159(1):1–11. doi: 10.1111/j.1095-8339.2008.00938.x

[34] Dunning LT, Savolainen V. Broad-scale amplification of matK for DNA barcoding plants, a technical note. Botanical Journal of the Linnean Society. 2010;164(1):1–9. doi: 10.1111/j.1095-8339.2010.01071.x

[35] Hamilton M. Four primer pairs for the amplification of chloroplast intergenic regions with intraspecific variation. Mol Ecol. 1999;8(3):521–3. 10199016

[36] Prince LM. Plastid primers for angiosperm phylogenetics and phylogeography. Appl Plant Sci. 2015;3(6):apps.1400085. doi: 10.3732/apps.1400085 26082876 PMC4467757

[37] Shaw J, Lickey EB, Beck JT, Farmer SB, Liu W, Miller J, et al. The tortoise and the hare II: relative utility of 21 noncoding chloroplast DNA sequences for phylogenetic analysis. Am J Bot. 2005;92(1):142–66. doi: 10.3732/ajb.92.1.142 21652394

[38] Katoh K, Standley DM. MAFFT multiple sequence alignment software version 7: improvements in performance and usability. Mol Biol Evol. 2013;30(4):772–80. doi: 10.1093/molbev/mst010 23329690 PMC3603318

[39] Ronquist F, Teslenko M, van der Mark P, Ayres DL, Darling A, Höhna S, et al. MrBayes 3.2: efficient Bayesian phylogenetic inference and model choice across a large model space. Syst Biol. 2012;61(3):539–42. doi: 10.1093/sysbio/sys029 22357727 PMC3329765

[40] Darriba D, Taboada GL, Doallo R, Posada D. jModelTest 2: more models, new heuristics and parallel computing. Nat Methods. 2012;9(8):772. doi: 10.1038/nmeth.2109 22847109 PMC4594756

[41] Rambaut A, Drummond AJ, Xie D, Baele G, Suchard MA. Posterior Summarization in Bayesian Phylogenetics Using Tracer 1.7. Syst Biol. 2018;67(5):901–4. doi: 10.1093/sysbio/syy032 29718447 PMC6101584

[42] Minh BQ, Schmidt HA, Chernomor O, Schrempf D, Woodhams MD, von Haeseler A, et al. IQ-TREE 2: New Models and Efficient Methods for Phylogenetic Inference in the Genomic Era. Mol Biol Evol. 2020;37(5):1530–4. doi: 10.1093/molbev/msaa015 32011700 PMC7182206

[43] Hoang DT, Chernomor O, von Haeseler A, Minh BQ, Vinh LS. UFBoot2: Improving the Ultrafast Bootstrap Approximation. Mol Biol Evol. 2017;35(2):518–22. doi: 10.1093/molbev/msx281 29077904 PMC5850222

[44] Yu G, Smith DK, Zhu H, Guan Y, Lam TT. ggtree: an r package for visualization and annotation of phylogenetic trees with their covariates and other associated data. Methods Ecol Evol. 2017;8(1):28–36. doi: 10.1111/2041-210x.12628

[45] Morrone JJ. Biogeographical regionalisation of the Neotropical region. Zootaxa. 2014;3782:1–110. doi: 10.11646/zootaxa.3782.1.1 24871951

[46] GBIF.org. GBIF Occurrence data download; n.d. [cited 2021 May 4]. 10.15468/dl.j26tht

[47] Natural Earth. Natural Earth: free vector and raster map data at 1:10m, 1:50m, and 1:110m scales, downloadable public comain dataset. n.d. [cited 2025 Jul 24]. https://www.naturalearthdata.com/features/

[48] Morrone JJ, Escalante T, RodrÍguez-Tapia G. Mexican biogeographic provinces: Map and shapefiles. Zootaxa. 2017;4277(2):277–9. doi: 10.11646/zootaxa.4277.2.8 30308652

[49] Gobierno de Estado de Tamaulipas. Acuerdo gubernamental por medio del cual se aprueba la actualización del programa de manejo del área ecologica protegida “Reserva del la Biósfera El Cielo”, ubicada en los municipios de Gómez Farías, Llera, Jaumave y Ocampo en el Estado de Tamaulipas, establecida mediante decreto gubernamental publicado el 13 de julio del 1985 (Anexo). Periodico Oficial del Estado de Tamaulipas. 2013;CXXXVIII(44):1–74.

[50] Pennington TD, Styles T, Taylor DAH. Meliaceae. New York: New York Botanical Garden. 1981.

[51] Calderón de Rzedowski G, Ramírez MT. Familia Meliaceae. Pátzcuaro: Instituto de Ecología. 1993.

[52] Germán-Ramírez MT. Meliaceae. Cd. de México: Universidad Nacional Autónoma de México. 2007.

[53] Rose JN. A new spanish cedar from central mexico. Contr U S Natl Herb. 1905;8:314–5.

[54] Finch KN, Jones FA, Cronn RC. Genomic resources for the Neotropical tree genus Cedrela (Meliaceae) and its relatives. BMC Genomics. 2019;20(1):58. doi: 10.1186/s12864-018-5382-6 30658593 PMC6339301

[55] León HWJ. Anatomía de la madera de 13 especies del orden Sapindales que crecen en el estado Mérida, Venezuela. Acta Botánica Venezuelica. 2006;29:269–96.

[56] Koecke AV. Spatio-temporal evolution of Cedrela (Meliaceae). Johann Wolfgang Goethe-Universität Frankfurt am Main. 2015. https://publikationen.ub.uni-frankfurt.de/frontdoor/index/index/year/2015/docId/37674

