# Supplementary material for: Integrative taxonomy of Cedrela (Meliaceae) leads to the recognition of a new species (C. tamaulipana) and the reinstatement of C. saxatilis
Source: PLoS One. 2025 Sep 17;20(9):e0329846. doi: 10.1371/journal.pone.0329846 (PMC12443259; doi:10.1371/journal.pone.0329846)
Supplement: S1 File — (DOCX) [file pone.0329846.s001.docx]

Appendix 1. GenBank accession numbers, voucher information and geographic origin of Meliaceae samples used in phylogenetic analysis. Newly generated sequences indicated with an asterisk (*).

| Taxon | Voucher | Origin/Source | *accD* | *matK* | *psbA-trnH* | *psbB-T-N* | *rbcL* | *rpl16* | *rpoB* | *rpoC1* | *trnS-G* | *ITS* |
| --- | --- | --- | --- | --- | --- | --- | --- | --- | --- | --- | --- | --- |
| *Azadirachta indica* A.Juss. | Samuel 5 (WU) | Sri Lanka | — | AY128180 | — | FJ462555 | AY128215 | — | — | — | FJ462522 | AY695594 |
| *Cedrela angustifolia* DC. | Pennington et al. 1150 (K) | Bolivia | HM368466 | — | KM408369 | FJ462541 | — | — | HM368500 | HQ291767 | FJ462509 | FJ462479 |
| *C. angustifolia* | Wood et al. 19222 (K) | Bolivia | HM368465 | — | KM408357 | FJ462540 | — | — | HM368499 | GU295825 | FJ462508 | FJ462478 |
| *Cedrela balansae* C.DC. | Zapater & Castillo 2406 (K) | Argentina | HM368460 | — | KM408358 | FJ462537 | — | — | HM368494 | GU295823 | FJ462505 | FJ462473 |
| *Cedrela cubensis* Bisse | Cuba excursion 462, Goethe  University Frankfurt/Main (FR) | Cuba | — | — | KM408408 | — | — | KM526693 | — | — | — | KM408337 |
| *Cedrela dugesii* S.Watson | German et al. 450 (K) | Mexico | HM368470 | — | KC155965 | FJ462545 | — | KM526683 | — | GU295828 | FJ462513 | FJ462483 |
| *Cedrela fissilis* Vell. | Agra et al. 5014 (K) | Brazil | HM368459 | — | KM408379 | FJ462535 | — | — | HM368493 | GU295821 | FJ462503 | FJ462471 |
| *C. fissilis* | Bolson 487 | Peru | — | KF555388 | KF421082 |  | KF561907 | — | — | — | — | KF421008 |
| *C. fissilis* | Muellner 2064 (K) | Peru | HM368462 | — | KM408380 | FJ462539 | — | — | HM368496 | GU295824 | FJ462507 | FJ462475 |
| *Cedrela kuelapensis* T.D.Penn. & Daza | Pennington et al. 17583 (K) | Peru | HM368458 | — | — | FJ462534 | — | — | HM368492 | GU295820 | FJ462502 | FJ462470 |
| *Cedrela molinensis* T.D.Penn. & Reynel | Pennington et al. 17765 (K) | Peru | HM368453 | — | — | FJ462530 | — | KM526687 | HM368487 | GU295817 | FJ462498 | FJ462466 |
| *Cedrela monroana* T.D.Penn. | Monro & Alexander 3081 (K) | El Salvador | HM368471 | — | KC155966 | FJ462548 | — | — | HM368504 | GU295831 | FJ462516 | FJ462486 |
| *Cedrela montana* Moritz ex Turcz. | Pennington et al. 17623 (K) | Peru | HM368467 | — | KC155972 | FJ462542 | — | — | HM368501 | GU295826 | FJ462510 | FJ462480 |
| *Cedrela nebulosa* T.D.Penn. & Daza | Pennington et al. 17646 (K) | Peru | HM368447 | — | KM408413 | FJ462524 | — | — | HM368481 | HQ291773 | FJ462492 | FJ462460 |
| *Cedrela oaxacensis* C.DC. & Rose | Mendoza et al. 291 (K) | Mexico | HM368469 | — | — | — | — | KM526686 | HM368503 | GU295827 | FJ462512 | FJ462482 |
| *Cedrela odorata* L. | Villacorta & Berendsohn 271 (K) | El Salvador | HM368456 | — | KM408366 | FJ462532 | — | — | HM368490 | GU295819 | FJ462500 | FJ462468 |
| *C. odorata* | Thuenen-ID CEODO_205_2  (Mader et al. 2018) | Cuba, population Guisa | Complete chloroplast genome MG724915.1 | | | | | | | | | — |
| *C. odorata* | Berrones-Morales 22 (IBUG 217369) | Mexico, Tamaulipas | OR230479* | OR230475* | — | OR230469* | OR230472* | OR230483* | — | — | OR230465* | — |
| *C. odorata* | Berrones-Morales 23 (IBUG 217370) | Mexico, Tamaulipas | OR230480* | OR230476* | — | OR230470* | OR230473* | OR230484* | — | — | OR230466* | PP752069* |
| *Cedrela* sp. | Neill et al. 6230 (K, MO) | Ecuador | HM368451 | — | KM408396 | FJ462528 | — | KM526705 | HM368485 | GU295816 | FJ462496 | FJ462464 |
| *Cedrela saltensis* M.A.Zapater & del Castillo | Zapater 2348 (K) | Argentina | HM368449 | — | KM408410 | FJ462526 | — | — | HM368483 | GU295815 | FJ462494 | FJ462462 |
| *Cedrela salvadorensis* Standl. | Formoso 2 (K) | Costa Rica | HM368472 | — | — | FJ462546 | — | — | HM368505 | GU295829 | FJ462514 | FJ462484 |
| *Cedrela saxatilis* Rose | Beitel s.n. (OSC OSC-V-258305), cited as CEOD-NYBG in Finch et al. (2019), Accession  683/89; cultivated in Bronx, NY, USA | Mexico, Oaxaca | Complete chloroplast genome without GenBank accession | — |  |  |  |  |  |  |  |  |
| *Cedrela tamaulipana* sp. nov. | Berrones-Morales 1 (IBUG 214565) | Mexico, Tamaulipas | OR230478* | MT877011* | MT877013* | OR230468* | MT877012* | OR230482* | — | — | OR230464* | — |
| *C. tamaulipana* | Berrones-Morales 26 (IBUG 217371) | Mexico, Tamaulipas | OR230481* | OR230477* | — | OR230471* | OR230474* | OR230485* | — | — | OR230467* | PP752068* |
| *C. tamaulipana* | Berrones-Morales 27 (IBUG 217372) | Mexico, Tamaulipas | — | PP754873* | — | PP754877* | PP754874* | PP754875* | — | — | PP754876* | — |
| *Cedrela tonduzii* C.DC. | Styles 82 (K) | Costa Rica | — | — | KM408388 | FJ462547 | — | — | — | GU295830 | FJ462515 | FJ462485 |
| *Cedrela weberbaueri* Harms | Daza 4012 (K) | Peru | HM368464 | — | — | FJ462536 | — | KM526700 | HM368498 | GU295822 | FJ462504 | FJ462472 |
| *C. weberbaueri* | Pennington et al. 17901 (K) | Peru | — | — | KC155974 | — | — | KM526699 | — | — | — | KM408340 |
| *Swietenia macrophylla* King | Chase 250 (NCU) | USA | — | EF489114 | — | FJ462554 | U39080 | EF489188 | — | — | FJ462521 | DQ861609 |
| *Toona ciliata* M. Roem. | PIF25085, AQ606666 (K) | Australia | HM368473 | — | — | FJ462549 | — | — | HM368506 | GU295832 | FJ462517 | FJ462488 |
