# Supplementary material for: Integrative taxonomy of Cedrela (Meliaceae) leads to the recognition of a new species (C. tamaulipana) and the reinstatement of C. saxatilis
Source: PLoS One. 2025 Sep 17;20(9):e0329846. doi: 10.1371/journal.pone.0329846 (PMC12443259; doi:10.1371/journal.pone.0329846)
Supplement: S2 File — (DOCX) [file pone.0329846.s002.docx]

Appendix 2. Selected specimens (Exsiccatae) of Mesoamerican *Cedrela.*

Specimens examined by the authors are marked with an exclamation mark symbol (!) following the herbarium acronyms, in accordance to Thiers (2018). Type status is abbreviated as follows: HT = holotype, LT = lectotype, IT = isotype. URLs are provided for key specimens available in online databases.

1. ***Cedrela discolor*** S.F.Blake, Proc. Biol. Soc. Washington 33: 108. (1920).

- Collection: *Palmer, E. 184*

MEXICO: Durango, San Ramon.

HT: US! [US-571205, barcode: 00108138] (Inflorescence).

<http://n2t.net/ark:/65665/37f61e10a-e48d-4345-94af-ac9ce2f61d03>

IT: GH! [GH-00044724] (Inflorescence).

<https://ids.lib.harvard.edu/ids/view/2551304>

Other IT: CM! [CM-213963]; F! [F-212958]; K! [K-000700276]; MO! [MO-1897727]; NY! [NY-01104712]; P! [P-01819660]; S! [S-08-12671]; US! [US-571204, barcode: 00108129].

*2.* ***Cedrela dugesii*** S.Watson, Proc. Amer. Acad. Arts XVIII: 190. (1882-83).

- Collection: *Dugès s.n.*

MEXICO: Guanajuato, Presa de la Olla.

LT: GH! [GH-00295521] (Inflorescence).

<https://plants.jstor.org/stable/viewer/10.5555/al.ap.specimen.gh00295521>

IT: A! [A-00044725]; GH!; MEXU!; NY! [NY-00659035].

- Collection: *Machuca 1275*

MEXICO: Jalisco, Municipio Jocotepec, Cerro Viejo (Volcano), cauce exposicion SW 2-3 km al Este de Huejotitlan.

USF! [USF-247440].

3. ***Cedrela monroana*** T.D.Penn., Monogr. Cedrela 27–31, fig. 2, map. (2010).

- Collection: *Monro, A.K., Alexander, D. 3081*

EL SALVADOR: La Libertad, Finca la Giralda, 5km before Comasagua.

HT: BM! [BM-000614297] (Inflorescence).

<https://plants.jstor.org/stable/viewer/10.5555/al.ap.specimen.bm000614297>

IT: B! [B-10-0037055]; BM! [BM-000600585]; K!; LAGU!; MO! [MO-795920]; NY!; US!.

- Collection: *Martínez, V.M 265*

EL SALVADOR: Santa Ana, San José Ingenio, P.N. Montecristo, cerro Los Cántaros.

B! [B-10-1244124] (Inflorescence).

<https://herbarium.bgbm.org/object/B101244124>

LAGU! [CMC00265]; BM!; MEXU!; MO! [MO-1016884].

- Collection: *Monro, A.K., Monterossa S., J., & Carballo R.A. 3789*

EL SALVADOR: Santa Ana, San José Ingenio, P.N. Montecristo, cerro Los Cántaros.

B; BM! [BM-000826047], ITIC, LAGU! [AM-3789], MO! [MO-801431].

- Collection: *Galán, P. 6026*

EL SALVADOR: Santa Ana, Mpio. Metapán, Ctón. El Limo, Crío. Las Mesas, propiedad de Pedro Madrid.

B; LAGU! [PG-06026]; MO.

4. ***Cedrela oaxacensis*** C. DC. & Rose, Contr. U.S. Natl. Herb. 5: 190. (1899).

- Collection: *Andrieux, G. 483*

MÉXICO: Oaxaca, Près d'Oaxaca.

HT: K! [K-000700281] (Inflorescence).

<https://plants.jstor.org/stable/viewer/10.5555/al.ap.specimen.k000700281>

IT: G-DC.

- Collection: *Pringle 4802*

MEXICO: Oaxaca, Monte Alban.

A! [A-00072402], B! [B-10-0248767], BM! [BM-000624701], CM! [CM-213722]; GH! [GH-000072401]; F! [F-106399], MEXU! [MEXU-16634], MO! [MO-1718335], PH! [PH-00003972]; S! [S09-218]; US! [US-237067, barcode: 00108168].

5. ***Cedrela odorata*** L., Syst. Nat., ed. 10. 2: 940. (1759).

- LT: P. Browne, Civ. Nat. Hist. Jamaica 158, t. 10, f. 1 (1756)! LT designated by C.E. Smith, Fieldiana, Bot. 29(5): 314 (1960). (Illustration, inflorescence & fruit).

JAMAICA.

<https://www.biodiversitylibrary.org/item/42074#page/527/mode/1up>

- - Collection: *Franck, A.R. et al. 3879*

JAMAICA: Middlesex Co., Clarendon Par., Peckham Woods, 4 km SW of B3 junction at Cave Valley.

USF! [USF-280149] (Fruit).

<https://cdn.plantatlas.org/img/specimens/USF/280149.jpg>

- - Collection: *Berrones-Morales 22 & 23*

MEXICO: Tamaulipas, Municipio de Gómez Farías

IBUG!.

6. ***Cedrela salvadorensis*** [Standl., Publ. Field Mus. Nat. Hist., Bot. Ser. 4: 215. (1929).](https://www.ipni.org/n/51023-2)

- Collection: *Calderón, S. 1007*

EL SALVADOR: Cerca de Chalchuapa, Cedro Macho.

HT: US! [US-1151973, barcode: 00108154] (Fruit).

<http://n2t.net/ark:/65665/m31514fe79-d73f-4540-8752-11369e63f2ef>

IT: US! [US-1151974**,** barcode: 01108342] (Fruit).

<http://n2t.net/ark:/65665/m3cd183f64-a300-4bee-80b0-0fd9cb4b47aa>

- Collection: *Martínez,* *V. M. 28*

EL SALVADOR: Depto. Santa Ana. San José Ingenio, P.N. Montecristo, la posa del coral.

MEXU! [MEXU-1187751] (Fruit).

<https://datosabiertos.unam.mx/IBUNAM:MEXU:1187751>

- Collection: *Conzatti, C. 3922*

MEXICO: Oaxaca; Portillo de Coyula, Distrito de Cuicatlán, Oaxaca.

US! [US-1080771, barcode: 00912520] (Inflorescence).

<http://n2t.net/ark:/65665/m396915879-2437-49db-b02b-c97e90447373>

7. ***Cedrela saxatilis*** Rose, Contr. US Natl. Herb. 8: 314. (1905).

- Collection: *Rose, J.N. & Painter, J.H. 6950*

MEXICO: Morelos, Cerca de Cuernavaca.

HT: US! [US-450517, barcode: 00108156] (Inflorescence).
<http://n2t.net/ark:/65665/m3e83ea507-c248-431b-81a6-fb0946c81723>

IT: US! [US-450516, barcode: 00108155] (Inflorescence).

<http://n2t.net/ark:/65665/m36a6f8967-f6e0-47b4-b0ee-1830929aa22a>

IT: K! [K-000700274] (Fruit).

<https://plants.jstor.org/stable/viewer/10.5555/al.ap.specimen.k000700273>

Other IT: GH! [GH-00072410]; MEXU! [MEXU-00016633]; NY! [NY-00053510].

- Collection: *Beitel, J. s.n.*

Cultivated at New York Botanical Garden, Nolan Greenhouse/08/I. Origin: MEXICO: Oaxaca.

OSC! [OSC-V-258305, {5E74C084-5003-4D81-9981-068D430C2407}, accession 683/89].

<https://oregonflora.org/collections/individual/index.php?occid=4205795>

8. ***Cedrela tamaulipana*** sp. nov., published in this work.

- Collection: *Berrones-Morales 1*

MEXICO: Tamaulipas, Municipio de Gómez Farías, Alta Cima, 6.81 km al Noroeste de Gómez Farías y 1.08 km al Oeste de Alta Cima.

HT: UAT! [UAT-22868] (Inflorescence & fruit).

IT: IBUG! [IBUG-214565] (Inflorescence& fruit).

- Collection: *Berrones-Morales 26*

MEXICO: Tamaulipas, Municipio de Gómez Farías, 5 km NE [from Gomes Farias].

IBUG! [IBUG-217371].

- Collection: *Berrones-Morales 27*

MEXICO: Tamaulipas, Municipio de Gómez Farías, 5 km NE [from Gomes Farias].

IBUG! [IBUG-217372].

- Collection *Gallardo-Yobal 145*

MEXICO: Tamaulipas, Municipio de Gómez Farías, 5 km NE [from Gomes Farias].

UAT! [UAT-22998].

9. ***Cedrela tonduzii*** C. DC., Bull. Herb. Boissier ser. 2. 5: 427. (1905).

- Collection: *Tonduz, A. 11945*

COSTA RICA: Copey.

IT: US! [US-00108151] (Inflorescence).
http://n2t.net/ark:/65665/m3e0a53f71-bbe7-4ecb-aa27-fa75f10a56da

- HT: CR; other IT: BM! [BM-000624700]; BR! [BR-522732]; G! [G-00016843]; GH! [GH-00044731]; K! [K-000700297]; NY! [NY-00053511]; US! [US-365956, barcode: 00921490].
